# Design and implementation of an interactive, competency-based pilot pediatric telemedicine curriculum

**DOI:** 10.1080/10872981.2021.1911019

**Published:** 2021-04-01

**Authors:** Marguerite Costich, Laura Robbins-Milne, Edith Bracho-Sanchez, Mariellen Lane, Suzanne Friedman

**Affiliations:** Department of Pediatrics, Division of Child and Adolescent Health, Columbia University Irving Medical Center, USA

**Keywords:** Telemedicine, curriculum, primary care, pediatrics, residents

## Abstract

During the height of the COVID-19 pandemic, telemedicine visits surged to increase access and maintain continuity of care, while reducing transmission of disease. However, few curricula exist for training residents on how to care for patients via telemedicine, especially in pediatrics. We aimed to create and evaluate an interactive, competency-based pilot curriculum, to meet the urgent need to train residents in telemedicine. The curriculum was developed in 2020 and includes a didactic, cased-based discussions, and direct observation exercise. A model for precepting residents, adhering to new ACGME guidelines, was also created to further engage residents in telemedicine in the outpatient general pediatrics settings. To evaluate the curriculum, we assessed feasibility of a direct observation to provide feedback and we conducted pre and post surveys to assess for changes in residents’ self-reported skills in performing telemedicine visits following implementation of the curriculum. 16 residents participated in the curriculum and 15 completed both the pre and post surveys (93%). Residents’ self-reported efficacy in performing key components of telemedicine visits, including completion of telemedicine visit (p = 0.023), initiation of visits (p = 0.01), and documentation (p = 0.001) all improved significantly following implementation. Residents’ perception of patient satisfaction with telemedicine and personal perception of ease of use of the telemedicine system increased, though neither were statistically significant. Uptake of the direct observation exercise was nearly universal, with all but one resident having a direct observation completed during their ambulatory month. This novel, interactive telemedicine pilot curriculum for residents addresses ACGME competencies and provides residents with a toolkit for engaging in telemedicine.

## Introduction

As New York City became the COVID-19 epicenter in March 2020 and in person visits were rapidly transitioned to telemedicine, there was an urgent need to train residents in basic principles of telemedicine and develop a model for precepting per new ACGME supervision guidelines [1]. To our knowledge, there is no literature describing a pediatric-focused telemedicine curriculum and a model for resident precepting in the pediatric ambulatory primary care setting [10].

We developed a multimodal, competency-based curriculum to train pediatric residents in telemedicine that includes a review of basic principles, case-based discussions, and a direct observation skills checklist. We evaluated the feasibility of the curriculum and changes in residents’ self- reported skills.

## Methods

A formal needs assessment was not conducted given the pressing need to train residents. Literature review and guidance from telemedicine content experts guided development of educational goals and objectives [2,3]. Training materials were created using institutional telehealth training materials and actual patient cases [4.] No additional materials, costs or time were incurred for the creation of the curriculum.

The pediatrics residency program at NewYork-Presbyterian Morgan Stanley Children’s Hospital includes 78 residents. All pediatric residents on their yearly, month-long ambulatory rotation participated in the curriculum. The three sessions were delivered via Zoom. The first session reviewed telemedicine basic principles, including how to join, set-up, document, and bill for a video visit and perform key components of the physical exam. The second and third sessions featured case-based sessions in an attending facilitated ‘morning report’ format. In addition, residents shared interesting and challenging cases that they had seen and groups discussed ways to optimize telemedicine functionalities and factors influencing clinical decision making and disposition. Examples of curriculum learning objectives and associated ACGME core competencies are provided in Table 1 [5.]

**Table 1.** Examples of cases presentation, associated learning objectives, and ACGME competencies

Case Presentation	Learning Objective	ACGME Competency
2-week old male infant with ‘vomiting’	Assess hydration status during a telemedicine encounter Differentiate physiologic vs. pathologic concerns	Patient Care
12 year old medically complex patient with chronic respiratory failure with trach/vent dependence, presents with ‘increased secretions.’	Learn how to use available resources (including home equipment) to appropriately assess and triage a medically-complex, technology-dependent patient during a telemedicine encounter	System-based Practice
3-week old male with ‘red bumpy rash’ and ‘new red spot near lip’	Learn how to instruct family to take photo of skin lesion and send to provider via MyChart. Learn how to view images sent from families in MyChart application.	Systems-based Practice
9 year old male with abdominal pain	Describe the components of an effective telemedicine abdominal and genitourinary exam. Assess when necessary to refer for in-person evaluation.	Patient Care Practice Based Learning
9 month female with medical complexity presents with concerns regarding medications.	Identify appropriate visit-types for telemedicine. Describe use of telemedicine to address health care coordination issues, such as access to subspecialists and medication reconciliation.	Practice Based Learning System-Based Practice
12 year old male with left hip pain	Describe the components of an effective telemedicine musculoskeletal exam. Assess when necessary to refer for in-person evaluation.	Patient Care Medical Knowledge

A direct observation (DO) telemedicine skills checklist was developed for the purposes of providing formative feedback. The skills on the checklist are reflective of multiple ACGME core competencies. The DO checklist was modeled on a DO utilized in training medical students in telemedicine and adapted for residents [6]. Particular emphasis was given on the checklist to nonverbal communication behaviors [7]. The DO was created and shared with the pediatric outpatient faculty prior to use. Adjustments to the tool were made based on feedback, providing content validity.

Our current precepting model utilizes the Epic (Epic Systems Verona, WI) ‘multi-provider’ video option, allowing multiple parties to join visits simultaneously. Residents and preceptors are present at the practice but in separate rooms, allowing for the residents to start their visit autonomously, followed by a conversation with the preceptor via EpicChat, in person or on the visit, with attendings joining the visit if needed. The debrief using the DO tool occurs after the visit.

Anonymous pre and post surveys were distributed via Qualtrics. Residents were surveyed on prior experiences using telehealth and ability to perform specific telemedicine-related skills prior to and after the completion of the curriculum on a 5-point Likert scale (1 = Strongly agree, 5 = Strongly disagree). Residents were also asked to indicate their level of agreement with statements regarding the importance of telemedicine and the impact of the COVID-19 pandemic on patient and provider perceptions of telemedicine. This study was approved by the Columbia University Medical Center IRB.

## Results

A total of 16 residents participated in the entire telemedicine curriculum. A total of 28 residents participated in the introduction to telemedicine didactic because their upcoming rotation required use of telemedicine and were administered the pre-survey. Eighteen residents completed the pre-survey, a response rate of 64%. The post-survey was administered only to those residents who had completed the full curriculum, with a response rate of 93% (n = 15).

Experience with telemedicine was limited prior to launching the telemedicine curriculum, with only 44% of residents having used telemedicine in any capacity. Experience with telemedicine for all residents increased after implementation of the curriculum, with more than half of respondents having completed more than ten visits.

Residents’ self reported skills in performing telemedicine visits improved significantly following the curriculum (Table 2). Residents’ perceptions of ease of use and patient satisfaction with telemedicine increased, though neither were statistically significant. Both before and after training, most residents agreed or strongly agreed that telemedicine is an increasingly common way to deliver care and that COVID-19 had changed their and their patients’ perceptions of its use.

**Table 2.** Resident self-reported skills pre and post curriculum

	Pre- Curriculum	Post-Curriculum	p
I can perform a telemedicine visit	2.55 (0.95)	1.67 (1.07)	**0.023**
I can recognize when use of a telemedicine visit may be an appropriate alternative to an in-person visit	1.94 (0.70)	1.67 (0.86)	0.34
I can appropriately triage a patient during a telemedicine visit	2.39 (0.59)	1.93(0.85)	0.10
I can initiate a telemedicine visit	2.83 (1.16)	1.80 (1.04)	**0.01**
I can document a telemedicine encounter	2.83 (0.95)	1.67 (0.83)	**0.001**
I can bill for a telemedicine encounter	3.78 (1.03)	2.87(1.45	0.06

(Scale: 1 = strongly agree, 2 = somewhat agree, 3 = neither agree or disagree, 4 = somewhat disagree, 5 = strongly disagree)

All but one resident (94%) who participated in the curriculum had a DO checklist completed. Scores for all skills observed were high, demonstrating acquisition and application of skill. The highest scores observed were in those skills not unique to the telemedicine encounter, such as appropriately diagnosing and triaging and clearly explaining treatment plan. (Table 3)

**Table 3.** Average direct observation score

Skill	Average Score (SD)
Ensures environmental privacy both for patient and provider.	2.82 (0.38)
Demonstrates ability to join visit on video visit platform	2.76 (0.54)
If needed, assists parents in joining visit on video visit platform. Can troubleshoot technical difficulties.	2.68 (0.68)
Creates note with correct verbiage needed for billing purposes	2.83 (0.37)
Uses appropriate professional telemedicine communication techniques (i.e., maintains simulated eye contact, uses natural speaking voice, remains within camera frame, uses visible hand gesturing)	2.94 (0.23)
Obtains appropriate history for visit needs	3 (0)
Conducts physical exam as appropriate and able, engaging patient/parent in the exam	2.77 (0.41)
Notes relevant observations from video of environment (e.g., relevant medical home equipment, medications, crib/home safety)	2.5 (0.5)
Provides family guidance on adjustments needed to video to improve visit (i.e., repositioning camera, ensuring adequate lighting)	2.68 (0.46)
Appropriate diagnosis and triaging based on patient information. Identifies when and how to escalate care (e.g., in-person office visit or referral to ED)	2.94 (0.22)
Offers clear explanation of treatment plan and next steps to family. Allows parent/patient to disconnect first.	2.94 (0.22)

(Scale: 1- needs improvement, 2– done, 3- done well)

## Discussion

Telemedicine will continue to serve an important role in clinical medicine post-pandemic. This is, to our knowledge, the first competency-based pediatric telemedicine curriculum for residents, combining logistical implementation, clinical reasoning and experiential learning. This curriculum directly addresses telemedicine-specific communication, physical exam and technical skills that have been identified as areas needing additional focus [8.] Following implementation of this curriculum, residents reported significantly improved self-assessed knowledge and skills in multiple telemedicine domains.

Many aspects of the curriculum are transferable to other specialties and programs. This curriculum uses limited resources and does not require substantial overhead costs. It can be easily and flexibly implemented with minimal adaptations in a variety of settings both within pediatrics and other subspecialties. Whereas prior curricula have used simulated telemedicine encounters with standardized patients, our curriculum emphasizes experiential learning and provision of formative feedback following observed telemedicine encounters with patients [9.]

One of the limitations of the curriculum is that only a small number of residents participated in this pilot curriculum. This study also lacks a control group which limits the potential impact of findings as residents’ skills may have improved simply from experience with telemedicine encounters and not the curriculum.

This pilot telemedicine curriculum provides a competency-based framework for teaching communication and clinical reasoning skills with just-in-time feedback that can be readily adapted to other specialties and programs and addresses an urgent need in primary care residency training.
